# Effects of Dietary Supplementation With *Saccharomyces cerevisiae* Yeast Extract on Production Performance in Aged Commercial Laying Hens

**DOI:** 10.1002/vms3.70517

**Published:** 2025-08-04

**Authors:** Ahmad Hassanabadi, Hassan Aalipour, Heydar Zarghi, Mohammadreza Salehan, Ramyar Gharedaghi

**Affiliations:** ^1^ Department of Animal Science Faculty of Agriculture Ferdowsi University of Mashhad Mashhad Iran

**Keywords:** egg quality, old laying hens, performance, *Saccharomyces cerevisiae*, yeast extract

## Abstract

**Background:**

Broken‐shell eggs in aged laying hens cause great losses to the egg industry. *Saccharomyces cerevisiae* yeast may improve shell quality by modulating intestinal health and oviduct function.

**Objectives:**

This study was carried out to determine the effects of supplementing *Saccharomyces cerevisiae* yeast extract (SYE) to the diets of aged commercial laying hens.

**Methods:**

A total of 192 Shaver White laying hens aged 105–116 weeks were allocated to four dietary levels of SYE (0, 1.0, 2.0, 3.0 g/kg diet), with 6 replicates of 8 birds each. The experiment was performed in a randomized complete block design.

**Results:**

The use of different levels of SYE caused a significant linear and quadratic increase in the percentage of laying and daily egg mass production (*p* < 0.05). Feed intake (FI) in the 3 g/kg treatment was significantly higher than the control treatment in 113–116‐week period (*p *< 0.05). The percentage of eggshell and shell thickness at 105–116 weeks of age (total period) in the 3 g/kg treatment was significantly higher than the control treatment (*p* < 0.05). Orthogonal comparison of the supplemented SYE against the control treatment also showed that the use of SYE in the diet significantly increased (*p* < 0.05) the percentage of shell (12.2% vs. 11.4%) and eggshell thickness (0.384 vs. 0.362 mm) compared to the control treatment.

**Conclusions:**

The use of different levels of SYE did not have a significant effect on production performance indicators in old laying hens of the Shaver White strain. It significantly increased the thickness and relative weight of eggshell. Supplementing SYE 3 g/kg diet significantly increased the average eggshell percentage and shell thickness.

## Graphical abstract

*Saccharomyces cerevisiae* yeast extract in the diet of laying hens did not have a significant effect on productive performance indicators. This extract increased the thickness and relative weight of eggshell.

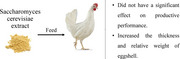

## 1 Introduction

Commercial laying hens productivity, like egg production and eggshell quality, and their overall health decline with age (Zhang et al. 2020; Bilal et al. 2022). Reduced shell quality and generally increased cracked eggs in the final stage of the laying cycle severely reduce the economic benefits for egg farmers and hinder the long‐term implementation of the laying cycle. To address these issues, nutritional strategies aimed at enhancing performance and extending the productive lifespan of laying hens have gained significant attention.

Nutritional adjustment strategies are widely recognized as positive tools for improving egg quality, especially eggshell (Mazzuco and Bertechini 2014; Cao et al. 2022). Ageing hens often have reduced intestinal calcium absorption, which is exacerbated by decreased activity of calcium‐binding proteins and hormonal changes affecting vitamin D metabolism, whereas oxidative stress from free radical accumulation impairs the function of the shell gland and further weakens structural integrity (Cao et al. 2022; Liu et al. 2023).

*Saccharomyces* is named from the words saccharo, meaning sugar and myces, meaning fungal plant. It is the most important industrial yeast for the production of biochemical products, recombinant proteins and single‐cell proteins and is known as baker's yeast (Stewart 2014). Yeast‐derived products have emerged as promising tools for improving animal health and productivity. *Saccharomyces cerevisiae* yeast extract (SYE), a byproduct of yeast fermentation, is rich in bioactive compounds such as nucleotides, amino acids, B vitamins and antioxidants. Previous studies have demonstrated the potential of SYE in improving performance and health in broilers and layers (Bilal et al. 2022), but its specific effects on aged commercial laying hens need further investigation.

Supplementation trials with SYE have shown increased egg production parameters. In a feeding study, 1.5–2.0 g/kg diet of *S. cerevisiae* to 40‐week‐old hens resulted in increased egg production and egg mass compared to controls. Paradoxically, although *S. cerevisiae* improved performance, its effect on shell thickness remains controversial. Hens fed 2.0 g/kg diet *S. cerevisiae* have reduced egg shell thickness (Hameed et al. 2019). Then, this study aimed to investigate the effect of dietary supplementation of SYE on productive performance and egg quality traits in aged laying hens at the age of 105–116 weeks.

## 2 Materials and Methods

### 2.1 Birds, Diets and Management

All procedures were approved by the Animal Care and Use Committee at the Ferdowsi University of Mashhad, Iran. In this experiment, a total of 192 Shaver White laying hens were used. The experimental period included a 2‐week pre‐experimental adaptation period from weeks 103 to 104 and a 12‐week recording period from weeks 105 to 116 of the laying period. This study was conducted in a randomized complete block design with four treatments, six replications and eight hens per replication. The basal diet was formulated on the basis of the Shaver White strain management guide for commercial layers (Shaver White commercial management guidelines; Table 1). SYE powder was supplemented to the basal diet at the doses of 0, 1.0, 2.0 and 3.0 g/kg during 105–116‐week of age for 12‐week. The chemical composition of the diet ingredients and the complete diet were determined on the basis of the AOAC (2019). The samples were ground and analysed for crude protein (Kjeldahl; *N* × 6.25; method 990.03), dry matter (DM; method 930.15) and total ash (method 942.05). The contents of calcium (Ca) and total phosphorus (P) in the diet were measured using Inductively Coupled Plasma‐Optical Emission Spectroscopy (ICP‐OES) instrument (Spectro Arcos, Kleve, Germany; method 968.08).

**TABLE 1:** Ingredients and nutrient composition of basal diet during 105–116 weeks; as fed basis.

Ingredients	(%)
Corn	52.3
Soybean meal (42% CP)	32.2
Wheat bran	0.3
Calcium carbonate	9.0
Soy oil	3.6
Dicalcium phosphate	1.4
Common salt	0.40
DL‐Methionine	0.2
NaHCO_3_	0.1
Vitamin premix^a^	0.25
Mineral premix^b^	0.25
Calculated values, %	
Metabolizable energy (kcal/kg)	2800
Crude protein	18
Calcium	3.83
Available phosphorous	0.40
Sodium	0.18
Methionine	0.49
Methionine + cystine	0.80
Lysine	1.0
Threonine	0.71
Determined values, %	
Dry matter	92.20
Crude protein	18.49
Ash	13.50
Calcium	3.46
Total phosphorous	0.47

aProvided per kg of diet: vitamin A (retinol), 8800 IU; vitamin D3 (cholecalciferol), 3300 IU; vitamin E (DL‐α‐tocopheryl acetate), 18.5 IU; vitamin K3 (menadione), 2.2 mg; vitamin B1 (thiamin), 2.2 mg; vitamin B2 (riboflavin), 5.5 mg; vitamin B3 (niacin), 28.0 mg; vitamin B5 (pantothenic acid), 6.6 mg; vitamin B6 (pyridoxine), 3.5 mg; vitamin B9 (folic acid), 0.7 mg; vitamin B12 (cyanocobalamin), 0.02 mg; biotin, 0.05 mg; antioxidant 1.0 mg.

bProvided mg/kg of diet: Mn (manganese sulphate) 80.0, Fe (iron sulphate) 75.0, Zn (zinc sulphate) 64.0, Cu (copper sulphate) 6.0, Se (sodium selenite) 0.3.

Cage dimensions were 60 cm length × 60 cm width × 40 cm height with a slope of 7.8 degrees. The cages were provided with two nipple drinkers and one trough feeder in front of the cages. During the experiment, birds received feed and water ad libitum. The lighting schedule in the house was 16 h of light with an intensity of 3.5 W per m^2^ of the house floor and 8 h of darkness. The temperature of the room was set in the range of 14–18°C. Each cage with eight birds was considered an experimental unit. Wood partitions were used to prevent cross‐feeding between the replicate cages.

### 2.2 Productive Performance

The laid eggs were collected daily at 7:00 AM and weighed using a digital scale (0.001‐g digital scale, model GF 400, A&D Weighing, San Jose, CA, USA). Egg production and the egg mass were calculated daily on the basis of the following formulas:

$$\begin{array}{ccc} \mathrm{Egg}\;\mathrm{production}\;\left(\%\right) & = & \mathrm{number}\;\mathrm{of}\;\mathrm{eggs}\;\mathrm{in}\;\mathrm{replicate}/\mathrm{number}\\ & & \mathrm{of}\;\mathrm{hens}\;\mathrm{in}\;\mathrm{replicate}\;\times 100 \end{array}$$

$$\begin{array}{ccc} \mathrm{Egg}\;\mathrm{mass}\;\left(g/\mathrm{hen}/\mathrm{day}\right) & = & \mathrm{egg}\;\mathrm{weight}\;\mathrm{in}\;\mathrm{replicate}\times \mathrm{egg}\\ & & \mathrm{production}\%\mathrm{in}\;\mathrm{replicate}/100 \end{array}$$

Feed intake (FI) was calculated at the end of each week, and the FI of each experimental unit during the entire experimental period was obtained from the average of 12 weeks. At the end of each week, the feed of each experimental unit was added to the specified bag after weighing and then gradually poured into the feeder of that unit. At the end of each week, after collecting the feed from the feeder, the amount of feed consumed was obtained from the difference in the weight of the feed added to the bags and the remaining feed. In all stages, the effect of the bag weight was applied in the calculations.

All the laid eggs were weighed for each replicate cage at the end of each week using a digital balance (0.001‐g, model GF 400, A&D Weighing, San Jose, CA, USA). The egg weight mean for each experimental unit over the entire experimental period was obtained from the average of 12 weeks.

The feed conversion ratio (FCR) was calculated by dividing the feed consumed (g) of each experimental unit by the weight of eggs produced (g) of the same experimental unit at the end of each week. The conversion ratio of each experimental unit during the entire experimental period was calculated by dividing the feed consumed (g) in each experimental unit during 12 weeks by the weight of eggs produced (g) in the same experimental unit during the period (Alsherify and Hassanabadi 2024). No mortality occurred during the experiment.

### 2.3 Egg Quality Traits

To measure quality traits at the end of each 28‐day period, three eggs were randomly selected from each experimental unit. The eggs were weighed using a digital balance (0.001‐g, model GF 400, A&D Weighing, San Jose, CA, USA). To measure egg specific gravity, a small mesh of the minimum size was made using copper wire and attached to the ring under the scale with a hook. The egg was completely immersed inside the mesh in a graduated beaker, and then the weight of the egg (excluding the weight of the mesh) was recorded as the weight in distilled water. It should be noted that the room and water temperatures were constant throughout the specific gravity measurement and the water was replaced at regular intervals. The following formula was used to calculate the specific gravity of the eggs:

$$\begin{array}{ccc} \mathrm{Specific}\;\mathrm{gravity} & = & \left(\mathrm{egg}\;\mathrm{weight}\;\mathrm{in}\;\mathrm{the}\;\mathrm{air}\right)/\left(\mathrm{egg}\;\mathrm{weight}\;\mathrm{in}\;\mathrm{the}\right.\\ & & \left.\mathrm{air}-\mathrm{egg}\;\mathrm{weight}\;\mathrm{when}\;\mathrm{submerged}\;\mathrm{in}\;\mathrm{water}\right) \end{array}$$

The specific gravity of the eggs in each experimental unit throughout the entire experimental period was obtained from the average of three 28‐day periods.

To measure egg shape index, three eggs at the end of each 28‐day period from each experimental unit were measured using a caliper with an accuracy of 0.01 mm (0.01‐mm digital caliper, model 1116‐150, Insize Co., Suzhou, China). Then, the shape index was calculated using the following formula (Card and Nesheim, 1972):

$$\mathrm{Shape}\;\mathrm{index}=\left(\mathrm{Egg}\;\mathrm{width}\;\left(\mathrm{mm}\right)\right)/\left(\mathrm{Egg}\;\mathrm{height}\;\left(\mathrm{mm}\right)\right)$$

The egg shape index in each experimental unit throughout the entire experimental period was obtained from the average of the three 28‐day periods.

To determine the percentage of internal components of eggs (yolk–albumen–shell), three eggs were randomly selected from each experimental unit at the end of each 28‐day period, and weight of yolk, albumen and shell was measured. For this purpose, the descriptive method of Prochaska et al. (1996) was used, in which the yolk and albumen were separated using a spoon for separating yolk and albumen, and as much excess albumen as possible was removed from the surface of the yolk using a paper towel. The weight of albumen was determined from the difference between the total weight of the egg and the sum of the weight of yolk and shell. The weight of the shell was also measured after washing with distilled water and exposure to air for 48 h. All weightings were performed using a digital balance (0.001‐g, model GF 400, A&D Weighing, San Jose, CA, USA). Then, the weight obtained for the yolk, white and eggshell was divided by the egg weight, and the result was expressed as a percentage of the total egg. The percentage of egg components of each experimental unit in the entire experimental period was obtained from the average of three 28‐day periods.

The height of the egg white was measured by a height gauge with an accuracy of 0.01 mm (0.01‐mm digital caliper, Insize Co., Suzhou, China). Then, it was calculated using the formula described by Haugh (1937) as follows:

Haughunits=100×log10−1.7×eggweightg0.37+7.57albumenheightmm−1.7×eggweightg0.37+7.57

The Haugh unit of each experimental unit in the whole experimental period was obtained from the average of three 28‐day periods.

The thickness of the eggshells was measured by a digital micrometre (0.01‐mm digital micrometre, model 293‐240, Mitutoyo Co., Kanagawa, Japan) from three points in the middle, top and bottom of the eggshell (Carter 1975). Then, the resulting average was considered the eggshell thickness. The eggshell thickness in each experimental unit in the entire experimental period was obtained from the average of the three 28‐day periods.

### 2.4 Statistical Analysis

The data were tested for normality using SAS 9.4 (SAS 2012) software through the univariate plot normal procedure. If the data were not normal, they were converted to arcsine for normalization and then subjected to statistical analysis. The data were statistically analysed using the GLM procedure, and the comparison of means was performed using the Tukey test at a probability level of 0.05. Orthogonal contrasts were performed between the groups receiving SYE and the group not receiving SYE.

## 3 Results

The use of different levels of SYE supplementation in the diet of aged Shaver White laying hens had no significant effect on egg production percentage. Orthogonal comparison of the groups receiving SYE against the control also did not show a significant effect on the egg production percentage of the laying hens in any of 28‐day experimental periods and the whole experimental periods. However, in the second period, egg‐laying percentage followed increasing linear trend with increasing levels of SYE (*p* = 0.05) (Table 2).

**TABLE 2:** Effect of dietary supplementation with *Saccharomyces cerevisiae* yeast extract (SYE) powder on the production performance of Shaver White laying hens at 105–116 weeks of age.

SYE (g/kg)	105–108 weeks	109–112 weeks	113–116 weeks	Total (105–116 weeks)
	**Egg production (%)**			
0 (control)	75.5	78.7	52.1	67.4
1	67.0	66.2	49.2	59.6
2	75.2	69.5	64.1	68.5
3	79.6	80.6	71.4	76.9
SEM	4.38	5.28	7.00	5.17
	*p* value			
Block	0.110	0.079	0.191	0.108
Treatment	0.258	0.192	0.123	0.169
Linear	0.313	0.050	0.973	0.298
Quadratic	0.185	0.065	0.451	0.123
Orthogonal (SYE vs. control)	0.781	0.300	0.233	0.880
	**Egg mass (g/bird/day)**			
0 (control)	47.7	49.1	32.4	42.1
1	43.3	41.5	31.2	37.5
2	48.1	45.4	41.1	44.3
3	49.5	51.9	45.3	48.9
SEM	3.13	3.13	4.59	3.31
	*p* value			
Block	0.301	0.141	0.207	0.329
Treatment	0.399	0.138	0.122	0.145
Linear	0.436	0.069	0.908	0.412
Quadratic	0.298	0.064	0.537	0.166
Orthogonal (SYE vs. control)	0.782	0.445	0.193	0.705
	**Egg weight (g)**			
0 (control)	63.3	62.8	62.2	62.8
1	63.1	62.6	63.1	62.8
2	64.1	65.7	64.0	64.8
3	62.1	64.4	63.4	63.5
SEM	1.10	0.945	0.761	0.740
	*p* value			
Block	0.211	0.178	0.091	0.108
Treatment	0.647	0.099	0.419	0.218
Linear	0.544	0.295	0.197	0.244
Quadratic	0.432	0.575	0.326	0.382
Orthogonal (SYE vs. control)	0.876	0.183	0.170	0.285

Dietary SYE levels had no a significant effect on egg mass production and egg weight. Orthogonal comparison of the groups receiving SYE against the control group also did not show a significant effect on these productive performance indices (Table 2).

FI was not significantly affected by dietary SYE during 105–108 weeks, 109–112 weeks and the whole experimental periods, but it was significantly affected in the third experimental period (113–116 weeks). So that, the SYE at a dose of 3 g/kg diet significantly increased FI compared to the control group (Table 3; *p* < 0.05). FI was not follow linear or quadratic trend in any of experimental periods. Orthogonal comparison of groups receiving SYE against the control also did not show a significant effect on the FI of aged laying hens (Table 3).

**TABLE 3:** Effect of dietary supplementation of *Saccharomyces cerevisiae* yeast extract powder (SYE) on feed intake, feed conversion ratio and cost of feed per kg of egg produced in Shaver White laying hens at 105–116 weeks of age.

SYE (g/kg)	105–108 weeks	109–112 weeks	113–116 weeks	Total (105–116 weeks)
	**Feed intake (g/bird/day)**			
0 (control)	144.2	122.1	133.9^a^	131.2
1	135.3	120.8	132.8^a^	128.9
2	136.8	124.2	138.0^b,a^	132.2
3	128.5	125.0	142.0^b^	132.5
SEM	7.46	2.51	2.05	1.89
	*p* value			
Block	0.306	0.145	0.254	0.340
Treatment	0.593	0.104	0.021	0.533
Linear	0.668	0.877	0.820	0.715
Quadratic	0.968	0.517	0.295	0.548
Orthogonal (SYE levels vs. control)	0.231	0.511	0.195	0.988
	**Feed conversion ratio**			
0 (control)	3.04	2.51	4.42	3.15
1	3.38	3.03	4.66	3.61
2	2.98	2.84	3.69	3.13
3	2.63	2.42	3.28	2.75
SEM	0.316	1.20	0.539	0.275
	*p* value			
Block	0.250	0.125	0.300	0.087
Treatment	0.439	0.148	0.275	0.218
Linear	0.490	0.042	0.952	0.286
Quadratic	0.297	0.064	0.530	0.133
Orthogonal (SYE vs. control)	0.912	0.282	0.372	0.974
	**Cost of feed/kg eggs produced ($)**			
0 (control)	1.15	0.95	1.68	1.20
1	1.29	1.16	1.78	1.37
2	1.14	1.09	1.41	1.20
3	1.01	0.93	1.26	1.06
SEM	0.122	0.067	0.204	0.099
	*p* value			
Block	0.360	0.130	0.607	0.361
Treatment	0.432	0.083	0.487	0.278
Linear	0.477	0.040	0.944	0.270
Quadratic	0.297	0.025	0.535	0.130
Orthogonal (SYE vs. control)	0.958	0.252	0.394	0.914

*Note*: Means without common superscript (a,b) within a column are significantly different (p < 0.05).

Feed cost per kg of egg produced was not significantly different among treatments (Table 3). Orthogonal comparison of SYE groups compared to the control group also showed no significant effect on feed cost per kg of egg produced in any of the 28‐day experimental periods and the entire experimental period. Linear and quadratic responses were significant only in the 109–112‐week period (Table 3).

FCR was not significantly affected by dietary SYE levels during any experimental periods. It does not show quadratic trend during any of experimental periods. FCR does not show a linear trend during 105–108 and 113–116 weeks and the whole experimental period. But it showed a linear response to SYE levers. Although the levels were increased, FCR was significantly decreased. Orthogonal comparison of groups receiving SYE against the control group did not show a significant effect on the FCR of aged laying hens (Table 3).

Different levels of SYE in the diet had no a significant effect on the egg specific gravity, egg shape index, albumen index, yolk percentage, albumen percentage and Haugh unit (Table 4). These indices also did not show a linear or quadratic response to dietary SYE levels. The use of SYE at a rate of 3 g/kg diet significantly increased (*p *≤ 0.05) eggshell percentage and its thickness compared to the control group. Orthogonal comparison of groups receiving SYE against the control group did not show significant differences in regard to egg specific gravity, egg shape index, albumen index, yolk percentage, albumen percentage and Haugh unit. But it revealed significant differences regarding to eggshell percentage (12.4% vs. 11.4%) and eggshell thickness (0.384 vs. 0.362 mm).

**TABLE 4:** Effect of dietary supplement of *Saccharomyces cerevisiae* yeast extract powder (SYE) on egg quality traits in Shaver White laying hens at 107–116 weeks of age.

SYE (g/kg)	Specific gravity (g/cm^3^)	Shape index (%)	Yolk (%)	Albumen (%)	Shell (%)	Shell thickness (mm)	Haugh unit	Albumen index (%)
0 (control)	1.08	78.8	27.6	61.0	11.4^a^	0.362^b^	88.8	83.6
1	1.09	78.1	28.3	59.3	12.4^c,b^	0.383^c,b^	91.1	80.0
2	1.09	78.7	27.1	60.4	11.6^b,a^	0.381^c,b^	89.2	78.4
3	1.09	77.9	29.6	61.2	12.6^c^	0.387^c^	90.7	80.5
SEM	0.002	0.686	0.747	57.7	0.270	0.008	1.78	2.59
	*p* value							
Block	0.251	0.089	0.240	0.590	0.270	0.490	0.134	0.130
Treatment	0.556	0.786	0.132	0.061	0.017	0.050	0.765	0.563
Linear	0.285	0.922	0.497	0.718	0.609	0.163	0.721	0.177
Quadratic	0.453	0.917	0.258	0.375	0.941	0.381	0.816	0.260
Orthogonal (SYE vs. control)	0.102	0.498	0.359	0.156	0.019	0.029	0.461	0.186

*Note*: Means without common superscript (a–c) within a column are significantly different (p < 0.05).

## 4 Discussion

In this study, there were no significant differences between the treatments in terms of production parameters and internal egg quality traits except for shell percentage which was improved by SYE supplementation. This result is in consistent with the results of other researchers (Yang et al. 2025; Sittiya and Nii 2024, 2025) and eggshell thickness which was improved by SYE supplementation. This finding is in consistent with the results of other studies (Sittiya and Nii 2024; Yang et al. 2025; Bozkurt, Küçükyilmaz, et al. 2012; Bozkurt, Tokuşoğlu, et al. 2012). It is generally agreed that eggshell quality is inversely related to egg weight. The present results are not consistent with the results of two other studies (Yang et al. 2025; Bozkurt, Tokuşoğlu, et al. 2012) which showed that feeding mannan and oligosaccharides did not significantly increase shell weight. Yeast is a microecological product produced under specific fermentation conditions. It is well established that yeast‐derived products have potential effects on poultry welfare and production. A number of studies have claimed that the use of yeast in the diet is beneficial for laying hens (Özsoy et al. 2018; Thanapal et al. 2021; Bilal et al. 2022). It has been observed that yeast improves the internal and external structure of eggs (Mikulski et al. 2012). The results of Yalçın et al. (2010) and Gurbuz et al. (2011) showed that by feeding yeast‐derived products, productive traits were improved, and yeast positively affected egg weight, feed efficiency and FI of aged laying hens. Cai et al. (2016) supplemented yeast enriched with trace elements in the diet of brown laying hens from 52 to 56 weeks of age at levels of 500 or 1000 mg/kg and found that the enriched yeast improved egg production. The supplement reduced mortality and FCR and increased albumin height, egg weight, eggshell strength, yolk weight and Haugh unit. Hydrolysed brewer's yeast supplementation had beneficial effects on egg‐laying performance, egg quality, nutrient digestibility and gastrointestinal microflora of laying hens (Park et al. 2020).

Meanwhile, Yalçın et al. (2014) did not observe a significant effect on FCR of laying hens with 2 g/kg yeast culture supplement, which is consistent with the present study. Administration of 0.5, 1 and 2 g/kg yeast or 2 g/kg SYE doses for 12 weeks did not affect egg weight, daily egg production or FI of 23‐week‐old laying hens, which is almost consistent with the present study.

Brown Bowens laying hens showed poor FCR when their feed was supplemented with yeast cultures at concentrations of 1, 2 and 3 g/kg (Hewida et al. 2011). Furthermore, Zhang et al. (2020) reported that adding yeast to the diet did not significantly affect egg production, which is in consistent with the results of the present study. Adding mannan oligosaccharides to the diet did not improve egg production in laying hens and did not affect the poor eggshell quality caused by heat stress except for an increase in eggshell weight, which is consistent with the present study (Bozkurt, Küçükyilmaz, et al. 2012). Similarly, body weight, FI, egg production and weight, FCR, mortality, eggshell thickness, albumin and yolk indices and Haugh unit in laying quails were not affected by dietary treatments with dry brewer's yeast, which is consistent with the present study, except for FI (third period) and shell thickness at 3 g/kg yeast extract supplementation level (Yalçın et al. 2008). These differences may depend on the bird species and yeast type. Similar results were obtained with the inclusion of dry baker's yeast in the diet, with the difference that dried baker's yeast at these levels significantly increased egg weight at 4% and 8%. Eggshell thickness decreased significantly at the 20% level, which is inconsistent with the present study. This could be related to the low percentage of yeast extract used in the present study (Yalçın et al. 2009). In a study conducted on aged laying hens (Swain et al. 2011), it was observed that the additive effects of yeast and probiotic supplementation on egg quality traits. The results showed that shell thickness and eggshell percentage increased at 1.5 and 2 g/kg supplementation, like the present study where shell thickness and shell percentage were increased. Hassanein and Soliman (2010) investigated the effect of different levels of live yeast on egg components in an experiment conducted on laying hens. They observed that eggshell percentage and eggshell thickness improved, especially at a concentration of 0.8%, which is consistent with the present study.

In a study, red yeast was supplemented at 1, 2 or 4 g/kg to laying hens diet for 38 weeks, and the results showed that egg shape index, yolk colour and Haugh unit were significantly increased in compared to the antibiotic‐supplemented group (Kanmanee et al. 2022). In another study, selenized yeast supplements increased egg albumin weight (Zhou et al. 2025). Similarly, Swain et al. (2011) reported improved egg albumin when yeast was fed to laying hens. This increase in the Haugh unit and albumin height due to yeast supplementation may be due to a critical micronutrient present in yeast. However, Yalçın et al. (2014) found that yeast was ineffective in improving internal egg quality in terms of Haugh unit and albumin height score. Similarly, 3 g/kg yeast cell wall for 8 weeks in older laying hens had no effect on shell strength and thickness, egg yolk colour, albumin height and Haugh unit despite improved egg‐laying performance (Zhang et al. 2020). According to Meseret et al. (2012), egg shell thickness and Haugh unit remained unaffected by the consumption of yeast‐containing diets, which is consistent with the present study. In accordance with the results obtained in the present experiment, no significant differences in albumin height, Haugh unit and eggshell thickness were reported in the study by Park et al. (2020) in laying hens fed brewer's yeast. But other research studies reported a positive effect of yeast supplementation on yolk weight and albumin weight (Gong et al. 2025), eggshell weight and yolk weight (Hakami et al. 2025) and Haugh unit and yolk shape index (Özsoy et al. 2018). These differences could be due to variations in the type and form of feed ingredients and their percentage contribution in the diet and the source of yeast (live or killed yeast). Published results on the effect of yeast supplementation on laying hens are quite contradictory. Several investigators reported an increase in egg‐laying percentage and egg weight following administration of yeast levels ranging from 0.7 to 4 g/kg. In quails fed diets supplemented with 2 and 4 g/kg of yeast, it has been found that egg white and yolk increased and decreased, respectively. However, Martinez et al. (2018) observed that yeast used in the diet of laying hens decreased the percentage of albumin production and increased the percentage of yolk.

## 5 Conclusions

The use of different levels of SYE (0, 1, 2 and 3 g/kg of feed) did not significantly affect any of the production performance indices as well as feed cost per kg of produced eggs in aged White Shearer laying hens. At the same time, the percentage of egg‐laying and egg mass production showed a significant linear and quadratic increase. Treatment of SYE at 3 g/kg diet caused a significant increase in FI compared to the control group. The use of SYE at a rate of 3 g/kg diet caused a significant increase in the relative weight of the eggshell and its thickness compared to the control group.

## Author Contributions

**Hassan Aalipour**: investigation, data curation, software, formal analysis, writing – original draft. **Mohammadreza Salehan**: writing and review. **Heydar Zarghi**: review and editing **Ramyar Gharedaghi**: writing – original draft. **Ahmad Hassanabadi**: project administration, methodology, conceptualization, funding acquisition, writing – review and editing.

## Ethics Statement

The authors confirm that the ethical policies of the journal, as noted in the journal's author guidelines page, have been adhered to and the appropriate ethical review committee approval has been received. The authors confirm that they have followed EU standards for the protection of animals used for scientific purposes and feed legislation. As part of this experiment, all animal procedures and ethics considerations were performed following the Guide to the Care and Use of Agricultural Animals in Research and Teaching (FASS 2010). Moreover, this study was conducted according to the procedures established by the Iranian Ministry of Agriculture (Experimental Authorization No. ASRI‐2016‐95014).

## Conflicts of Interest

The authors declare no conflicts of interest.

## Peer Review

The peer review history for this article is available at https://www.webofscience.com/api/gateway/wos/peer‐review/10.1002/vms3.70517.

## Data Availability

The data that support the findings of this study are available from the corresponding author upon reasonable request.
